# Efficacy of convalescent plasma for the treatment of severe influenza

**DOI:** 10.1186/s13054-020-03189-7

**Published:** 2020-07-29

**Authors:** Zhiheng Xu, Jianmeng Zhou, Yongbo Huang, Xuesong Liu, Yonghao Xu, Sibei Chen, Dongdong Liu, Zhimin Lin, Xiaoqing Liu, Yimin Li

**Affiliations:** State Key Laboratory of Respiratory Diseases, Guangzhou Institute of Respiratory Health, First Affiliated Hospital of Guangzhou Medical University, Department of Critical Care Medicine, 151 Yanjiang Street, West Guangzhou, 510120 Guangdong China

**Keywords:** Efficacy, Convalescent plasma, Severe influenza, Meta-analysis

## Abstract

**Background:**

Convalescent plasma administration may be of clinical benefit in patients with severe influenza, but reports on the efficacy of this therapy vary.

**Methods:**

We conducted a systematic review and meta-analysis assessing randomized controlled trials (RCTs) involving the administration of convalescent plasma to treat severe influenza. Healthcare databases were searched in February 2020. All records were screened against eligibility criteria, and the risks of bias were assessed. The primary outcome was the fatality rate.

**Results:**

A total of 2861 studies were retrieved and screened. Five eligible RCTs were identified. Pooled analyses yielded no evidence that using convalescent plasma to treat severe influenza resulted in significant reductions in mortality (odds ratio, 1.06; 95% CI, 0.51–2·23; *P* = 0.87; *I*^2^ = 35%), number of days in the intensive care unit, or number of days on mechanical ventilation. This treatment may have the possible benefits of increasing hemagglutination inhibition titers and reducing influenza B viral loads and cytokine levels. No serious adverse events were reported. The included studies were generally of high quality with a low risk of bias.

**Conclusions:**

The administration of convalescent plasma appears safe but may not reduce the mortality, number of days in the intensive care unit, or number of days on mechanical ventilation in patients with severe influenza.

## Introduction

Seasonal and pandemic influenza cause substantial disease and a high economic burden [1]. The main treatment for influenza is neuraminidase inhibitor administration [2]. Despite this therapy, pandemic influenza remains a major cause of morbidity and mortality globally [1, 3, 4]. Therefore, there is a need for effective therapy against influenza. Convalescent plasma therapy is a promising option that has been used experimentally for the last 100 years, since the Spanish flu of 1917–1918, and is currently being tested as a potential treatment for the novel coronavirus, severe acute respiratory syndrome coronavirus-2 (SARS-CoV-2) [5–7].

Preclinical animal studies have demonstrated the therapeutic efficacy of hyperimmune immunoglobulin and IgG antibody from convalescent plasma [8, 9]. It has been suggested that the administration of high-titer anti-influenza immune plasma derived from convalescent or immunized individuals may be clinically beneficial for the treatment of seasonal and pandemic influenza [10–12]. Additionally, treatment with convalescent plasma was reported to reduce hospital stays and mortality in patients with SARS-CoV infection [10] and in patients with severe influenza A (H1N1) [13]. Some systematic reviews of studies using convalescent plasma concluded that there is evidence of clinical benefits in such patients [10, 14, 15].

Until recently, the collective evidence based on previous studies has been of relatively poor quality because very few randomized trials had been conducted. However, two randomized, controlled, and multicenter trials were reported in 2019, and in both trials, convalescent plasma or hyperimmune intravenous immunoglobulin (H-IVIG) prepared from pooled plasma, obtained from convalescent patients, and conferred no significant benefit over placebo in patients with influenza infection [16, 17]. This is not concordant with previous studies [13, 18]. To investigate this discrepancy, the current study conducted a systematic review and meta-analysis evaluating the clinical efficacy of either convalescent plasma or H-IVIG for the treatment of severe influenza.

## Materials and methods

### Inclusion and exclusion criteria

We conducted this study in compliance with the PRISMA guidelines [19]. Prospective randomized controlled trials (RCTs) involving patients with influenza who were treated with convalescent plasma and/or H-IVIG were considered for inclusion in the analysis. The reports considered for inclusion were limited to those published in English. Crossover trials, before-after studies, conference presentations, abstract publications, case reports or case series, studies with no comparator, and editorials were excluded from consideration.

### Search strategy

Two authors (ZH and JZ) performed the literature search during February 2020. To increase the sensitivity of the search, the search term “influenza” was used in conjunction with AND “convalescent plasma” OR “convalescent serum” OR “hyperimmune immunoglobulin” OR “immune plasma” OR “H-IVIG” as keywords or Medical Subject Heading (MeSH) search terms. The records of four electronic databases (PubMed, EMBASE, Scopus, and Web of Science), dating from their inception to February 10, 2020, were searched.

### Definitions

The study population of interest included severe patients of any age or sex who were hospitalized with laboratory-confirmed influenza infection (as defined in the original trials). Severe influenza was defined of having either hypoxia (room air oxygen saturation of < 93%) or symptoms of respiratory distress or using the authors’ definitions, including a National Early Warning (NEW) score of > 2 points or a CURB-65 (severity score for community-acquired pneumonia) score of > 3 points. The interventions of interest were convalescent plasma, serum, or H-IVIG derived from convalescent or immunized individuals. Comparator treatments included placebo and low-titer plasma.

### Outcomes

The primary outcome of interest in the current analysis was the influenza case-fatality rate. The secondary outcomes analyzed included antibody levels, cytokine levels, viral loads, incidences of serious adverse events, and numbers of days spent on mechanical ventilation, in the intensive care unit (ICU), and in the hospital.

### Data extraction

Two authors (ZX and JZ) independently reviewed the articles retrieved via the above-described search protocol and extracted the relevant data from them. Discrepancies were resolved via discussion.

### Quality assessment

The quality of each trial included in the analysis was assessed based on a thorough review of the details provided in the “Materials and Methods” section and any relevant supplementary materials. Trial quality was also assessed using the Cochrane collaboration tool for assessing the risk of bias [20], including assessment of random sequence generation, allocation concealment, blinding (of interventions and outcome measurement or assessment), incomplete outcome data, selective reporting bias, and other potential sources of bias (e.g., industry funding). For each criterion, the risk of bias was rated as low, high, or unclear in cases where there were insufficient details. Two authors (ZX and JZ) independently assessed the study quality, and disagreements were resolved via discussion.

### Assessment of heterogeneity

The *I*^2^ statistic was used to evaluate the influence of heterogeneity on the pooled results, and an *I*^2^ value of > 50% was deemed to indicate substantial heterogeneity [20]. Fixed-effects models were used to pool data when the level of heterogeneity was insignificant, and random effects models were used to pool data when significant heterogeneity was identified.

### Statistical analysis

Categorical data were pooled, and odds ratios (ORs) and 95% confidence intervals (CIs) were calculated. We did not construct funnel plots, as fewer than 10 trials were identified for each comparison. Statistical analyses were conducted using Review Manager software (version 5.3; Nordic Cochrane Centre, Cochrane Collaboration, Copenhagen, Denmark), and two-sided *p* values of < 0.05 were considered statistically significant.

## Results

### Description of studies

**Fig. 1:**
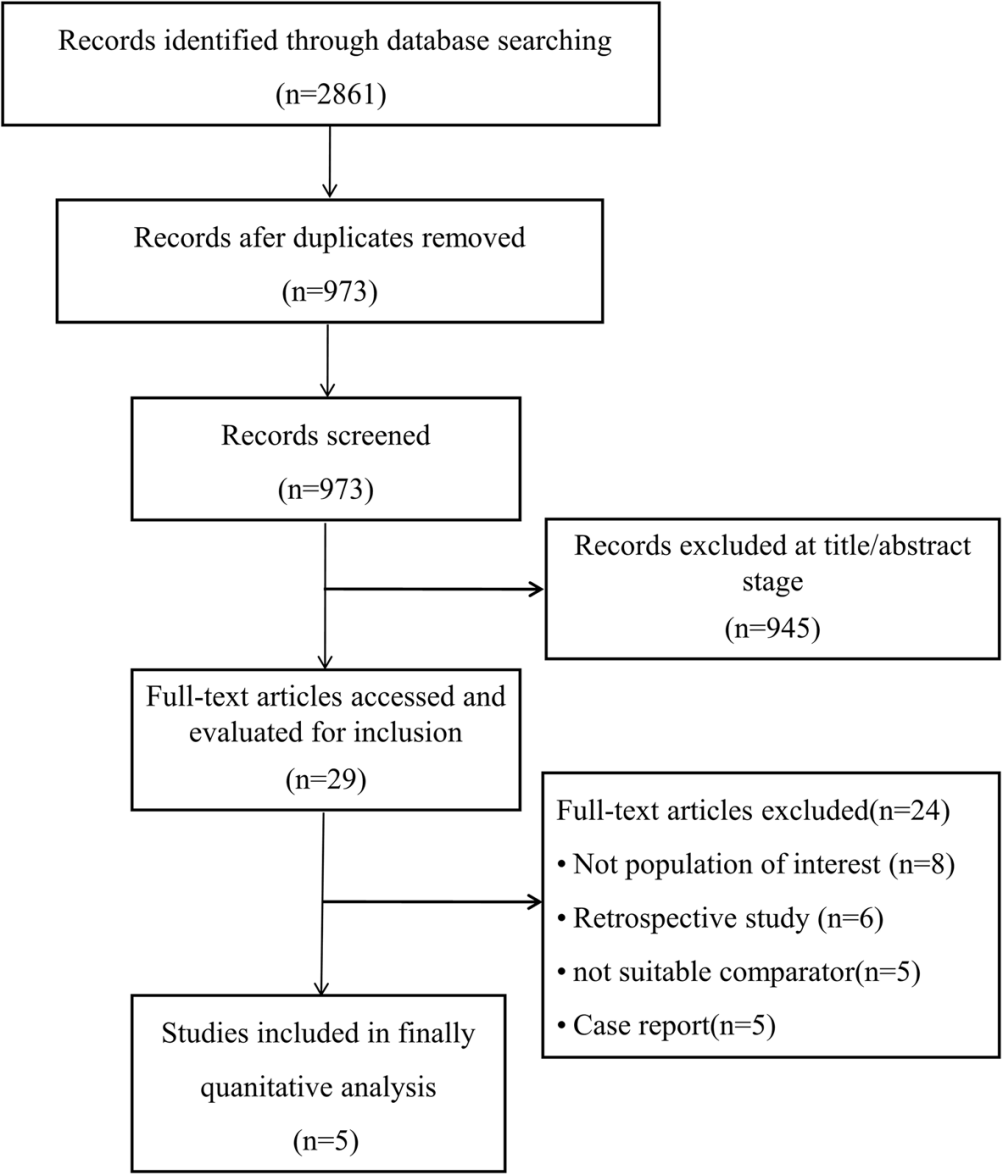
Search strategy used to identify reports for inclusion

The initial search identified 2861 potentially eligible reports. After the exclusion of duplicates and irrelevant articles, 29 trials were deemed to warrant further detailed review. Twenty-four of these reports were subsequently excluded because they did not meet the predefined eligibility criteria, ultimately resulting in the inclusion of five trials in the present analysis (Fig. 1).

All five studies included in the present analysis were randomized, controlled, and multicenter trials. Hung et al. [18] reported that H-IVIG administered within 5 days of symptom onset was associated with a lower viral load and less mortality in patients with severe H1N1 infection. In a pilot study reported by the INSIGHT FLU005 IVIG Pilot Study Group (Group IFIPS) [21], H-IVIG administration was associated with significantly higher hemagglutination inhibition (HAI) titers in patients with influenza. The same group subsequently performed an international double-blind RCT in which H-IVIG administration was associated with similar safety outcomes regarding death and adverse events [17]. In 2017, Beigel et al. [22] reported a multicenter phase 2 trial in which immune plasma was associated with non-significant reductions in the number of days in hospital for patients with severe influenza. More recently, however, their phase 3 trial indicated that high-titer anti-influenza plasma conferred no significant benefit in patients with severe influenza A [16] (Table 1).

**Table 1:** Characteristics of included studies

NO.	Author	Journal, years	Study design	Multi-center	Population	Dose	Treatment (*n*)	Control (*n*)	Outcomes
1	Hung, et al. [18]	CHEST, 2013	RCT, H-IVIG vs. normal IV immunoglobulin (IVIG)	Yes	Patients with severe H1N1 infection	0.4 g/kg	17	17	H-IVIG was associated with a lower viral load and reduced mortality
2	Group IFIPS [21]	The Journal of Infectious Diseases, 2016	RCT, H-IVIG vs. placebo	Yes	Patients with influenza A or B	0.25 g/kg	16	15	H-IVIG administration significantly increases HAI titer levels among patients with influenza
3	Davey Jr., et al. [17]	Lancet Respir Med, 2019	RCT, H-IVIG vs. placebo	Yes	Patients with influenza A or B infection	0.25 g/kg	156	152	H-IVIG was not superior to placebo for adults hospitalized with influenza infection
4	Beigel et al. [22]	Lancet Respir Med, 2017	RCT, immune plasma vs. standard care	Yes	Patients with severe influenza A or B	HAI titers ≥ 1:80	42	45	Immune plasma provided support for a possible benefit of severe influenza
5	Beigel et al. [16]	Lancet Respir Med, 2019	RCT, high-titer anti-influenza plasma (≥ 1:80) vs. low-titer (≤ 1:10)	Yes	Patients with influenza A	HAI titers ≥ 1:80	91	47	High-titer anti-influenza plasma conferred no significant benefit over non-immune plasma

### Risks of bias

**Fig. 2:**
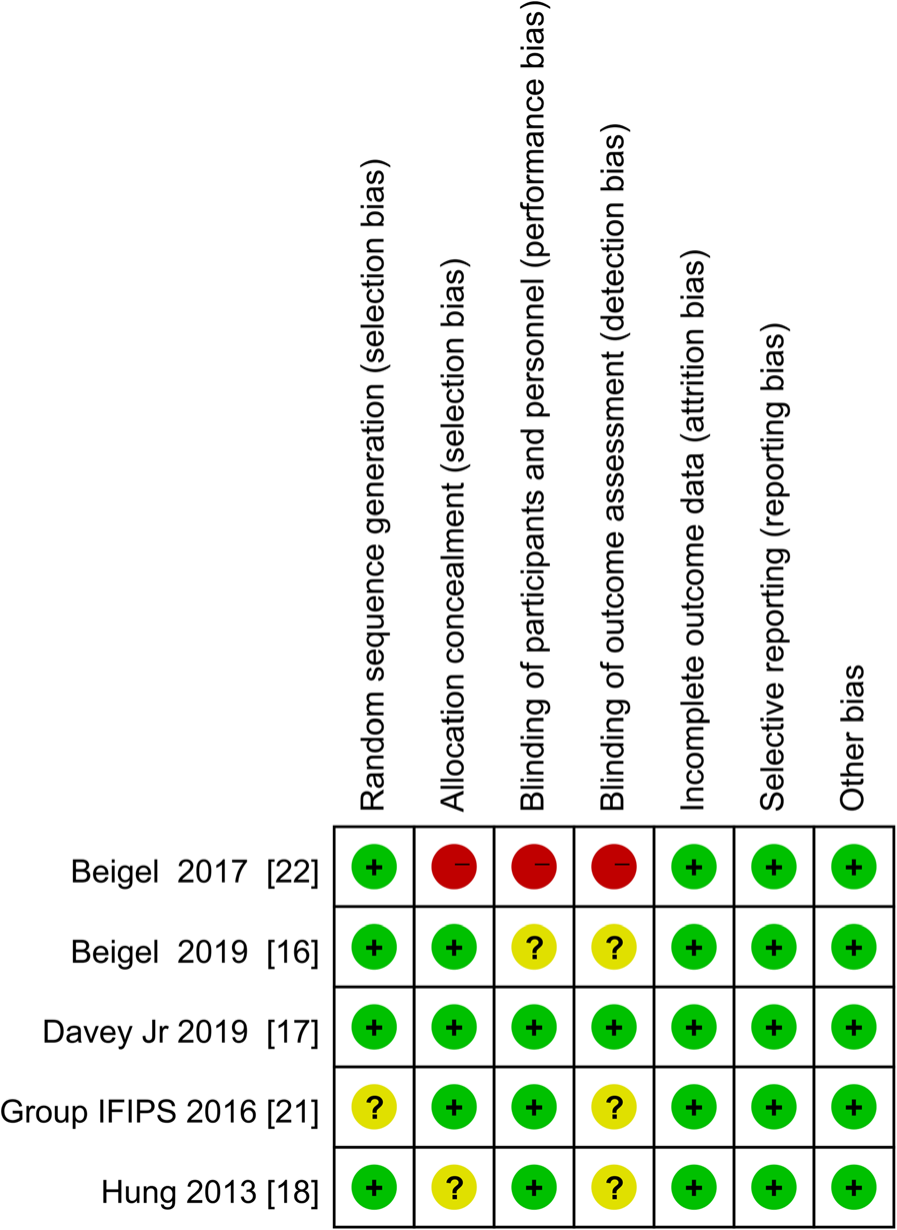
Diagram depicting the risks of bias in each study. Green represents low risk, yellow represents unclear risk, and red represents high risk

The RCTs included in the current analysis [16–18, 21, 22] were all deemed to have low risks of attribution bias, reporting bias, and selection bias, with the exception of the Group IFIPS study [21] in which the details pertaining to random sequence generation were unclear. The phase 2 trial reported by Beigel et al. [22] was an open-label study; consequently, no allocation concealment and blinding were performed, so the study has high risks of performance bias and detection bias. The subsequent phase 3 trial by this group [16] was a multicenter, randomized, double-blind study with low risks of attribution bias, reporting bias, and selection bias (Fig. 2).

### Mortality outcomes

**Fig. 3:**
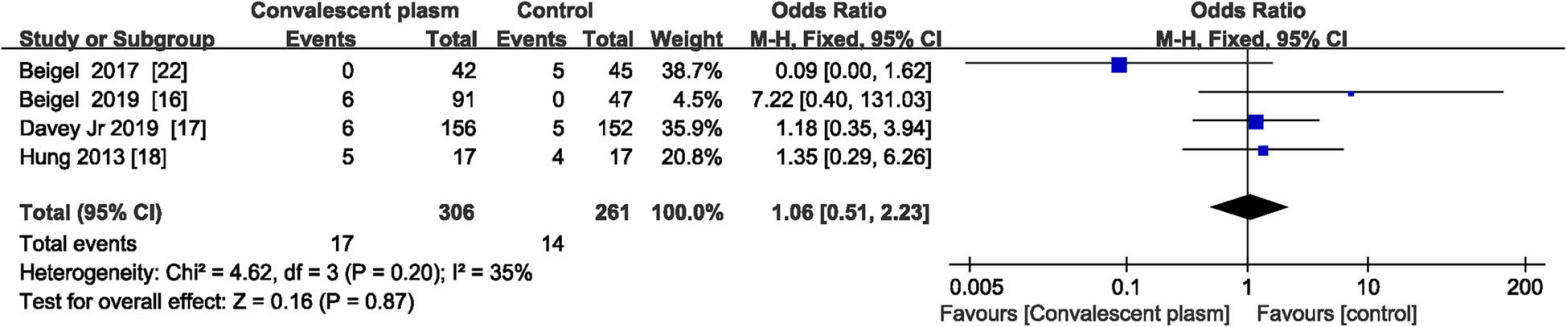
Pooled estimates of case-fatality rates due to severe influenza in patients who were administered convalescent plasma and in control patients

Four of the trials in the current study included extractable data facilitating an assessment of the efficacy of immune plasma/H-IVIG administration for reducing mortality from severe influenza [16–18, 22]. Based on an analysis of the pooled data (*n* = 567), there was no significant difference in mortality between patients with severe influenza treated with immune plasma/H-IVIG and those who received a placebo (OR = 1.06; 95% CI = 0.51–2.23; *P* = 0.87; *I*^2^ = 35%) (Fig. 3).

### Secondary outcomes

#### Antibody levels

It was reported that the HAI titers significantly increased in patients with influenza A or influenza B who received H-IVIG but that those increased titers gradually decreased after the first week of treatment [17, 21] (Table 2).

**Table 2:** Secondary outcomes

Secondary outcomes	Author	H-IVIG/immune plasma group	Control group	*P* value
Antibody levels	Davey Jr., et al., 2019 [17] Group IFIPS, 2016 [21]	Significantly increases HAI titer levels among patients with influenza A and B	–	–
Viral loads	Hung et al., 2013 [18]	3.3 log 10 copies/mL(H1N1)	4.67 log 10 copies/mL	0.04
	Davey Jr., et al., 2019 [17]	Mean log10 RNA − 1.95(Influenza A)	− 2.62	0.02
	Davey Jr., et al., 2019 [17]	Mean log10 RNA − 2.09(influenza B)	− 1.54	0.005
	Beigel et al., 2017 [22]	Median log 10 copies per mL 1.9 (1.9–1.9) day 7 (Nasal swab, Influenza A and B)	1.9 (1.9–1.9)	NS
Cytokines	Hung et al., 2013 [18]	TNF-a, IL-1ra, and IL-10 fell to a similar level as control 3 days after treatment	–	–
Mechanical ventilation, day	Beigel et al., 2017 [22]	0 (0–6) (influenza A and B)	3 (0–14)	0.14
	Beigel et al., 2019 [16]	9 (4–16) (influenza A)	15.5 (7.0–29.0)	0.22
Length of ICU stay, day	Hung et al., 2013 [18]	11 (4–13.5) (H1N1)	10 (4.5–13.5)	NS
	Beigel et al., 2017 [22]	2.5 (0.0–9.0) (influenza A and B)	3 (0–13)	0·37
	Beigel et al., 2019 [16]	5.0(3.0–12.5) (influenza A)	8 (4–25)	0.32
Length of hospital stay, day	Hung et al., 2013 [18]	16 (11.5–13.5) (H1N1)	16 (7–29)	NS
	Beigel et al., 2017 [22]	6 (4–16) (influenza A and B)	11 (5–25)	0·13
	Beigel et al., 2019 [16]	5 (3–12) (influenza A)	6 (4–12)	0.30
Serious adverse events	Beigel et al., 2017 [22]	20% (influenza A and B)	38%	0·041
	Beigel et al., 2019 [16]	35% (influenza A)	32%	NS

#### Viral loads and cytokines

Hung et al. [18] reported that the H1N1 viral loads were significantly lower in patients treated with a convalescent plasma infusion than in the control group subjects, as were the levels of cytokines interleukin-1ra, interleukin-10, and tumor necrosis factor alpha. However, in another large clinical trial, the reductions in overall viral load during the first 3 days did not differ significantly between the H-IVIG and placebo groups (*P* = 0.49) [17]. In that trial, 16% of the patients in the H-IVIG group and 20% of the patients in the placebo group had no detectable virus after infusion (*P* = 0.15). In the subgroup of patients with influenza B, the decline in viral loads appeared greater in the H-IVIG group than in the placebo group, but this difference did not reach statistical significance (*P* = 0.053) [17] (Table 2).

#### Length of ICU and overall hospital stay

Both Hung et al. [18] and Beigel et al. [16, 22] reported that there were no significant differences in the length of ICU stay or overall hospital stay between an H-IVIG/immune plasma treatment group and a control group. In the Beigel et al. study [16, 22], there was also no significant difference in the number of days on mechanical ventilation between an immune plasma treatment group and a standard care alone group. In the Davey et al. study [17], there were no significant differences in the proportions of patients alive and discharged at days 7 and 28 between an H-IVIG group and a control group (Table 2).

#### Serious adverse events

No adverse events related to treatment were reported by Hung et al. [18] or Davey et al. [17]. In the open-label RCT reported by Beigel et al. [22], there were fewer serious adverse events in the participants who were administered an immune plasma infusion than there were in the control group subjects; however, in the subsequent double-blind trial by the same group [16], there were similar numbers of serious adverse events in both groups, the most frequent of which was acute respiratory distress syndrome (Table 2).

## Discussion

The current analyses suggest that convalescent plasma may not have clinically relevant effects on mortality in patients with influenza. Reductions in the number of days in the ICU, overall hospital stay lengths, and the number of days on mechanical ventilation following treatment with convalescent plasma were also not significant. Of interest, there was evidence of a possible benefit from this therapy by way of increased HAI titers and reduced influenza B viral loads and cytokine levels after convalescent plasma treatment. No serious adverse events were reported.

The use of immune plasma has been recommended as a primary therapy in patients with severe respiratory infectious diseases including influenza, severe acute respiratory syndrome, and Middle East respiratory syndrome [10, 14, 22]. However, until recently, relevant data pertaining to these recommendations were weak and limited to case reports and case series lacking controls. Compared with the previous meta-analyses [10, 14, 15], our meta-analysis differs in the inclusion criteria utilized, in the number of trials included, and in the summary estimates of treatment effect, which were strengthened by an extensive search, duplicate citation screening, and data abstraction. We focused on high-quality RCTs and estimated not only fatality rates but also both the biological effects (i.e., HAI titers, viral loads, cytokines) and clinical benefits (i.e., length of ICU/hospital stays, number of days on mechanical ventilation, and adverse events). The evidence for a reduction in mortality associated with convalescent plasma was strongest for influenza A (H1N1) [18], but this should be interpreted with an appropriate degree of caution because of the limited sample size (*n* = 17) and the early use of treatment (onset within 5 days) in that study. Additionally, in an analysis of pooled data derived from four trials (*n* = 567) in which deaths were reported, there was no significant association between the use of convalescent plasma and mortality in patients with severe influenza.

With regard to secondary outcomes, including the number of days in the ICU, overall number of days in the hospital, and the number of days on mechanical ventilation, three RCTs reported relevant data, and the reductions in an H-IVIG/immune plasma group compared with a control group were not significant in any of them [16, 18, 22]. Despite robust increases in the HAI titers against influenza A and B [17, 21], reductions in the influenza B viral loads [17], and reductions in the cytokine levels in patients with H1N1 [18], no clinical benefit of receiving H-IVIG/immune plasma infusion was evident in influenza patients.

Our meta-analysis has some limitations. First, despite an extensive literature search, we identified only four trials with a primary outcome that could be pooled. Second, the severity of influenza may have been different between the evaluated RCTs. Third, we did not registering in PROSPERO, but we conducted this study in compliance with the PRISMA guidelines [19]. Finally, we were not able to pool all data reported for outcomes such as viral loads, cytokine levels, and ICU and hospital stay lengths, due to variability in the measuring and reporting of these outcomes.

Presently, many questions remain about the use of convalescent plasma for treating influenza. For example, it is still unknown how much of severe disease is due to virus replication versus inflammation. The composition of plasma is complex, and transfusion reactions can occur after the administration of blood products [23, 24]. Furthermore, titers of the relevant antibodies contained in convalescent serum preparations differ. The standardized extraction and purification of specific antibodies can be difficult and time-consuming. Lastly, viral shedding and the induced immune responses may be different between influenza A and B. Thus, more definitive animal and pilot studies should be conducted to identify the optimal timing, dosage, and indications for the use of H-IVIG/immune plasma in patients infected with different virus subtypes.

## Conclusion

The available high-quality evidence suggests that convalescent plasma/H-IVIG is safe but unlikely to reduce mortality in patients with severe influenza. Further clinical trials with larger populations remain needed to evaluate the efficacy of convalescent plasma for the treatment of severe influenza.

## Data Availability

The datasets used and/or analyzed during the current study are available from the included randomized controlled trials.

## Abbreviations

COVID-19: Coronavirus disease 2019
CIs: Confidence intervals
Group IFIPS: INSIGHT FLU005 IVIG Pilot Study Group
H-IVIG: Hyperimmune intravenous immunoglobulin
HAI: Hemagglutination inhibition
ICU: Intensive care unit
MeSH: Medical Subject Heading
ORs: Odds ratios
RCTs: Randomized controlled trials
SARS-CoV: Severe acute respiratory syndrome coronavirus
